# Enhanced conflict monitoring via a short-duration, video-assisted deep breathing in healthy young adults: an event-related potential approach through the Go/NoGo paradigm

**DOI:** 10.7717/peerj.3857

**Published:** 2017-10-06

**Authors:** Kok Suen Cheng, Yun Fah Chang, Ray P.S. Han, Poh Foong Lee

**Affiliations:** 1 Department of Mechatronics & Biomedical Engineering, University Tunku Abdul Rahman, Bandar Sungai Long, Selangor, Malaysia; 2 Department of Mathematical & Actuarial Sciences, University Tunku Abdul Rahman, Bandar Sungai Long, Selangor, Malaysia; 3 College of Engineering, Peking University, Beijing, China

**Keywords:** Mindfulness practices, Event-related potential, Practice duration, Conflict monitoring, Go/nogo task, Deep breathing

## Abstract

**Objectives:**

Practitioners of mindfulness are reported to have greater cognitive control especially in conflict monitoring, response inhibition and sustained attention. However, due to the various existing methods in each mindfulness practices and also, the high commitment factor, a barrier still exists for an individual to pick up the practices. Therefore, the effect of short duration deep breathing on the cognitive control is investigated here.

**Methods:**

Short duration guided deep breathing videos consisting of 5, 7 and 9 min respectively were created and used on subjects training. The effect on cognitive control was assessed using a Go/NoGo task along with event-related potential (ERP) measurements at Fz, Cz, and Pz.

**Results:**

From the study, the significant outcome showed at the follow-up session in which participants engaged for 5 min deep breathing group showed a profound NoGo N2 amplitude increment as compared to the control group, indicating an enhanced conflict monitoring ability. An inverse relationship between the NoGo N2 amplitude and the breathing duration is observed as well at the follow-up session.

**Conclusion:**

These results indicated the possibility of performing short duration deep breathing guided by a video to achieve an enhanced conflict monitoring as an alternative to other mindfulness practices and 5 min is found to be the optimum practice duration.

**Significant:**

This study is the first to establish a relationship between deep breathing and conflict monitoring through ERP. The study population of young adults taken from the same environment reduces the variance in ERP results due to age and environment.

**Limitation:**

A larger sample size would provide a greater statistical power. A longer duration of deep breathing should be investigated to further clarify the relationship between the practice duration and the NoGo N2 amplitude. The result can be split by gender and analyzed separately due to the different brain structure of males and females.

## Introduction

Humans possess the ability to flexibly change their behaviors according to the current situation, reflecting the need to maintain the goals for that particular situation. This ability, known as cognitive control, is vital in everyday life given that our behaviors need to be altered accordingly when facing unknown situations (Davidson et al., 2006). Among the different cognitive processes that build up the cognitive control, *conflict monitoring* and *response inhibition* are of most interest due to their relevance to mental disorders. Conflict in the cognitive sense occurs when two or more incompatible processes compete for the same attentional resources simultaneously (Botvinick et al., 2001). One example of conflict would be response overriding, whereby a prepotent response towards a normal stimulus needs to be inhibited. After the conflicting information has been monitored then the part of response inhibition comes into play so that the prepotent response can be successfully inhibited. A third component that comes into play is the sustained attention, defined as the long-term placement of one’s attention on a particular task or process (Sarter, Givens & Bruno, 2001). Sustained attention is characterized by the need to be vigilance enough to pick up and react to any unexpected stimuli or conflicts and thus, modulates the process of conflict monitoring and response inhibition (Barkley, 1997).

Conflict monitoring and response inhibition are needed in a Go/NoGo task which normally consists of two stimuli; a majority Go stimulus that requires a certain action, whether covert or overt, and a minority NoGo stimulus that requires no actions (See et al., 1995). The commission error would reflect in these processes as a successful inhibition would lead to lower commission error (Roche et al., 2005). Furthermore, given the huge skewing of probability towards the Go stimulus, the norm would be in responding to the majority of Go stimulus and an autopiloting action might come in Robertson et al. (1997). The mind will start to wander around instead of being fully focused on the task (Robertson et al., 1997; Manly et al., 1999) and this act of mind-wandering will reduce the performance in the task (Thomson, Besner & Smilek, 2015). Besides conflict monitoring and response inhibition, the Go/NoGo task also requires sustained attention and is indexed by the omission error and reaction time variability (O’Halloran et al., 2011). To further elucidate the cognitive process during this task, event-related potentials (ERP) are employed due to its high temporal resolution. In the context of the Go/NoGo task, two components are elicited, namely the N2 and P3 components for each Go and NoGo trials. The NoGo N2 and P3 are used to reflect the conflict monitoring and cancellation of preplanned response towards the Go stimulus, respectively (Donkers & Van Boxtel, 2004; Smith, Johnstone & Barry, 2007), whereas the Go P3 can serve as a biomarker for the sustained attention level (Datta et al., 2007; Hart et al., 2012).

Practitioners of mindfulness are shown to have greater sustained attention, conflict monitoring and response inhibition compared to control groups with no experience in mindfulness (Zeidan et al., 2010; Teper & Inzlicht, 2013; Quaglia, Goodman & Brown, 2016). By definition, mindfulness is the placement of one’s attention to the present moment and accepting any thoughts that occur without any judgment. An operational definition of mindfulness given by Bishop et al. (2004) suggests that mindfulness can be separated into two facets: self-regulation of attention and orientation of one’s experience in the present moment. Sustained attention is required for the first facet such that the mind is kept in the present and would not wander around whereas for the second facet, it involves an awareness and acceptance of any conflicts or judgmental thoughts that are distracting. The first facet is directly relating to the cognitive control while the second facet of awareness and acceptance is also shown to be related to the cognitive control (Teper, Segal & Inzlicht, 2013). By practicing mindfulness, the cognitive control would be enhanced through improvements in the above two facets. Even though the benefits of mindfulness practices are established, there are some potential barriers that make the community reluctant to embrace such practices. One such barrier is the existence of different exercises being employed in each mindfulness practice. For example, Mindfulness-based Stress Reduction (MBSR) has the element of mindful walking whereas for the Zen meditation there is an element of Tandem Breathing, both of which are not present in the other practice. Besides, the high demand in time and the need for guidance from a coach can discourage the community from taking up these practices as well (Carmody et al., 2008). For a full MBSR course, it can take up to eight weeks, with weekly meetings that last for about three hours and daily practice of about an hour (Kabat-zinn, 1996). This can be difficult to adhere to especially in the current fast-paced lifestyle of young people.

Deep breathing is a common element in most mindfulness practices, for instance, yoga, tai chi, and meditation (Brown & Gerbarg, 2009). Recent interest has surged in the investigation of the effects of deep breathing on human cognition. The literature has reported the effects of deep breathing on the retention of newly learned motor skills (Yadav & Mutha, 2016) and the aging-related cognitive decline (Ferreira et al., 2015). Yadav & Mutha (2016) had provided the first evidence to link deep breathing to motor memory by showing that a 30-minute deep breathing intervention led to a greater performance in a motor skill. This better retention of the motor skill was evident both immediately after the breathing session, as well as, after a one-day break. The study by Ferreira et al. (2015) on the other hand, investigated the cognitive decline due to aging in three interventions, namely an aerobic exercise group, a respiratory training group, and a control group. The respiratory training group had undergone seven forms of respiratory training among 20 of them, in which one of them was the deep breathing. At the end of the study, the attention level (indexed by the Wechesler Adult Intelligence Symbol Search subscale) of the respiratory training group was stable showing no significant difference between the pre- and post-measurement, whereas for the control and aerobic exercise groups, there was a significant decline in the attention level. Combining the above results and the fact that deep breathing is a common element in many mindfulness practices, a possible linkage between deep breathing, conflict monitoring, and sustained attention is expected. A plausible mechanism underlying how deep breathing can lead to a better cognitive control is that deep breathing will alter the activation of the anterior cingulate cortex (ACC) which facilitates cognitive control (Sridharan, Levitin & Menon, 2008). A greater activation of the ACC would be required to resolve any conflicts such as distracting thoughts in order to maintain the attention placed on the deep breathing process (Holzel et al., 2011). This greater activation of the ACC, in turn, can be inferred from the ERP measurements obtained from a Go/NoGo task (Bokura, Yamaguchi & Kobayashi, 2001).

Published reports on mindfulness have considered these practices as a combination of different exercises and constant practices; however, the actual physiological effects that associate deep breathing with the cognitive control is still lacking. In this study, the effects of short duration deep breathing on the cognitive control, specifically the conflict monitoring, response inhibition, and sustained attention are investigated via behavioral and ERP results obtained from a Go/NoGo paradigm for both an immediate and a follow-up effect after seven days. Furthermore, the effects of different practice times are investigated in order to clarify and arrive at an optimum practice time. The deep breathing used in this study is of short durations (5, 7, and 9 min), which are in line with the required daily practice time of many mindfulness practices (Baer, 2014; Bower et al., 2015), and the participants are guided by a video. The sample size is restricted to young adults to reduce the variability in the ERP result due to age (Lucci et al., 2013). It was hypothesized that through deep breathing, there would be an increase in terms of the sustained attention indexed by a larger Go P3 amplitude, less reaction time variability, and omission error. Besides that, a better conflict monitoring and response inhibition are expected for the deep breathing group as indexed by a larger NoGo N2 and P3 amplitude along with less commission error. Lastly, the improvement of the above variables was hypothesized to be larger for greater deep breathing duration.

## Methods and Materials

### Participants

56 young adults (23% female, age range: 20–27), which comprised mainly of undergraduates, were recruited through the distribution of flyers. We have also adopted the following exclusion criteria: (1) those who have been sick for the immediate past two weeks, (2) having taken or are on medication or drug prescriptions and (3) those who experienced difficulties in deep breathing after 5 min or more. All participants had normal or corrected-to-normal vision, no respiratory diseases or psychiatric disorder as reported by themselves. There were five dropouts during the study, and thus, a total of 51 participants completed the protocol. In terms of the ethnic composition, 92% were Malaysian Chinese, 4% were Malaysian Indian, 2% were Ayran and 2 % were Sino-Kadazan. Details of the participants in each group are shown in Table 1.

**Table 1:** Demographic information of the participants. The values are represented in mean and standard deviation in brackets.

	Con (*n* = 13)	DB5 (*n* = 12)	DB7 (*n* = 13)	DB9 (*n* = 13)
Age (years)	22.00 (1.96)	22.17 (1.47)	22.23 (1.79)	21.85 (1.46)
Age range (years)	22–27	20–24	20–24	20–24
Height	168.8 (8.3)	166.4 (9.3)	171.3 (9.24)	169.4 (7.2)
Weight	61.3 (15.0)	59.1 (13.9)	65.9 (12.8)	59.7 (11.9)

### Deep breathing intervention

**Figure 1:**
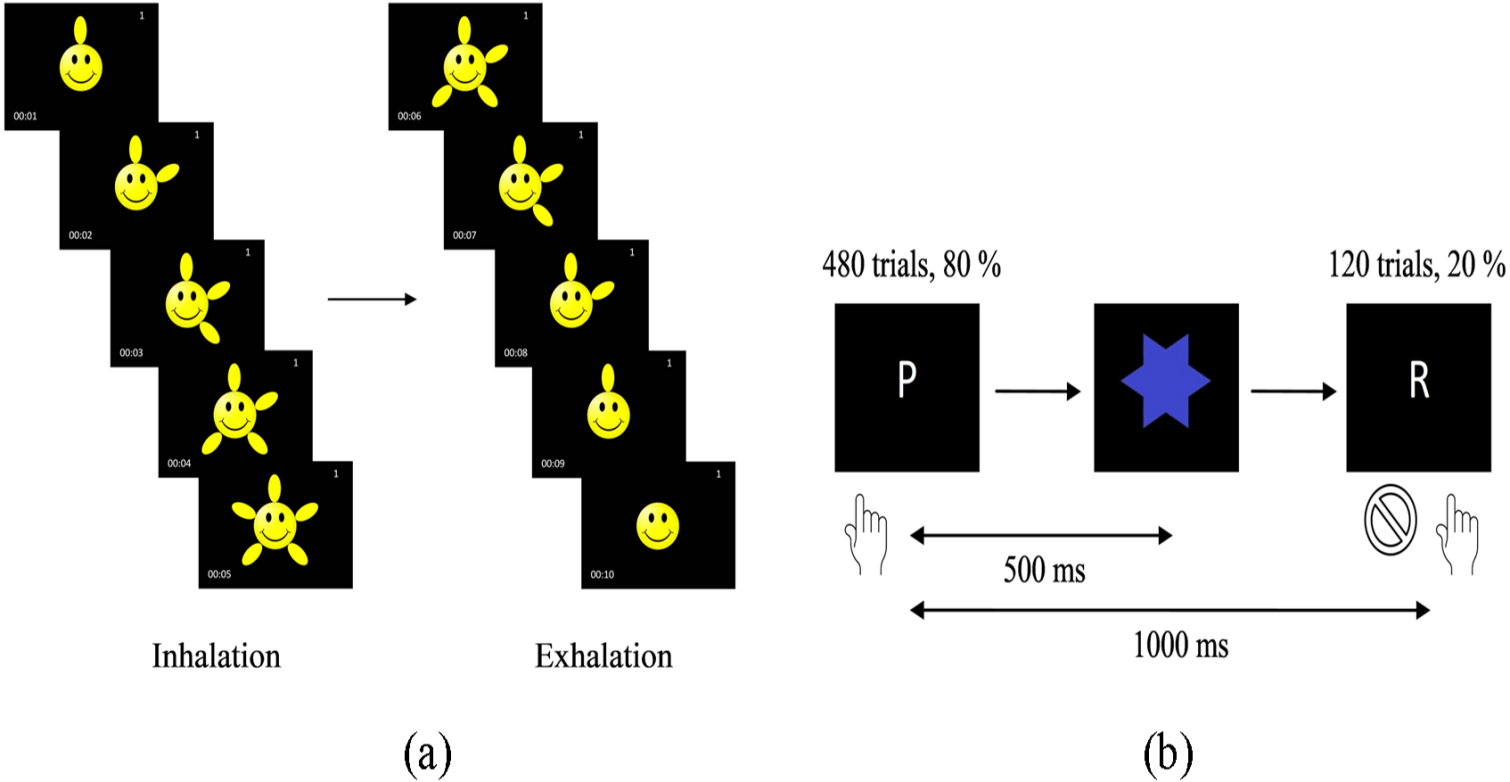
Screenshots of the guided deep breathing intervention video (A) and the Go/NoGo task (B). The breathing rate is set to six breaths per minute, with five seconds of inhalation when the petals appear and five seconds of exhalation when the petals disappear. Stimuli in the Go/NoGo task whereby ‘P’ need to be responded whereas ‘R’ does not need to be responded.

The breathing rate for the deep breathing (DB) group is set at six breaths per minute (Prakash et al., 2006; Critchley et al., 2015). The DB group are required to strictly observe the instructions shown in a video in order to achieve a specified breathing rate for the breathing duration of 5 min, 7 min and 9 min, respectively. In the video, there were appearing petals for the inhalation and disappearing petals for the exhalation process as depicted in Fig. 1A. The participants were instructed to keep their focus on the video, their breathing and also, to feel the air going in and out of their body.

### Task paradigm

In this study, the Go/NoGo task was implemented using The Psychology Experiment Building Language (PEBL; Mueller & Piper, 2014), which is an open source software. The two stimuli in this test were the letters ‘P’ and ‘R’, with a stimulus time of 500 ms, followed by a blue star for 500 ms and thus, an interstimulus time of 1,000 ms (see Fig. 1B). In total, there were 480 Go ‘P’s (80%) and 120 NoGo ‘R’s (20%). The stimuli were given in two separate blocks with 300 stimuli in each block with a short break between the blocks. The participants were required to click on the mouse pad whenever a ‘P’ appeared and to refrain from any action whenever an ‘R’ appeared. Prior to the test, there was a practice session with 20 stimuli (16 ‘P’s and 4 ‘R’s) for the participants to get familiar with the task. At the end of the task which lasted about 10 min, the overall accuracy (OA), Go reaction time (Go-RT), omission error (OE), commission error (CE) and reaction time variability (RTV) were recorded.

### Procedure

The research procedures were approved by the local university scientific and ethical review committee (Reference no: U/SERC/04/2017). The whole experimental procedures were explained to the participants and informed consent was obtained from all individual participants in the study. The participants were assigned into four groups: Control Group (Con) without the video guided deep breathing intervention, Deep breathing for 5 min (DB5), Deep breathing for 7 min (DB7) and Deep breathing for 9 min (DB9) without biased based on their arrival time slot for the experiment. However, there were four participants who did not come for the follow-up session and hence were removed from analysis. The final number of participants in each group were 13, 12, 13 and 13 for Con, DB5, DB7, and DB9, respectively.

The experiment was carried out in a room with an adequate lighting of two fluorescent lamps. On arrival to the laboratory, the participants were first instructed to rest for 15 min in a comfortable sitting position to ensure a stable physiological state was achieved. At the same time, the EEG equipment was applied to the participants. A baseline reading was taken in the next 5 min (R1). Immediately after the baseline reading, the participants performed the Go/NoGo task (T1). After finishing the test, the DB participants began the deep breathing intervention (INT) following the deep breathing video. For the control group, they were instructed to relax for 9 min and no instructions about their breathing or videos were given. After the intervention time (5 min, 7 min or 9 min), the participants rested for another 5 min (R2) before the Go/No-go task (T2) was repeated again. Throughout the recording, the participants were required to open their eyes and to not move. This concluded the first session.

**Figure 2:**
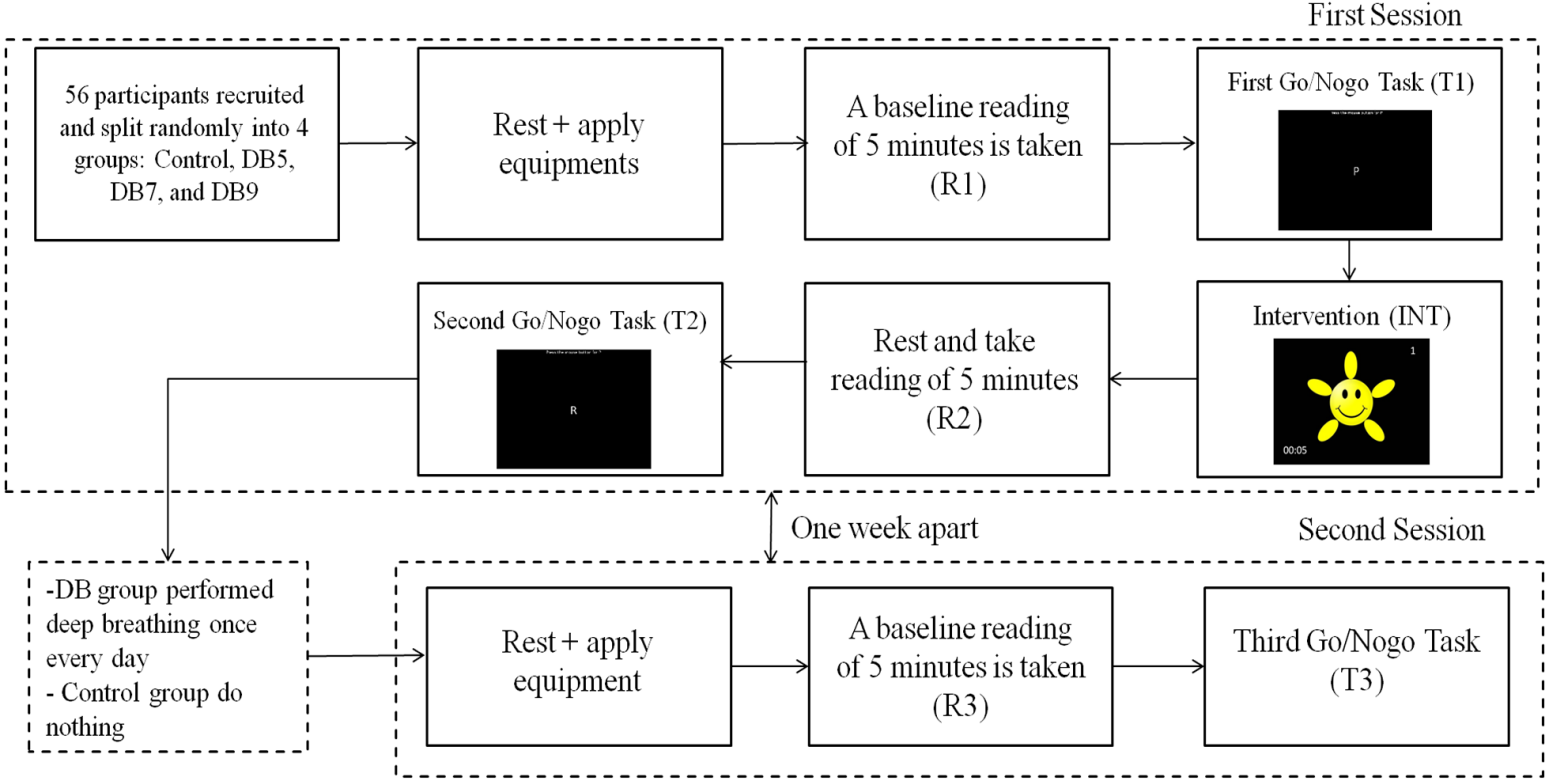
The whole experimental procedure which consists of two sessions that were one week apart. Between the first and second session, the DB groups were required to follow the video and perform the deep breathing once every day.

For the DB participants, they were required to perform the deep breathing intervention seven times (the deep breathing during the first session is considered as one), every day once at any time suitable for them. The deep breathing video clip was sent to the DB participants and a reminder was sent to the participants prior to the pre-agreed time slot, and after finishing the deep breathing exercise, the participants sent a confirmation to the researchers. As for the control group, these instructions were not given. All the participants returned exactly one week after their first session at the same timeslot. During the follow-up session, the baseline reading was again recorded for 5 min (R3). However, in this session, the Go/No-go task was only performed once after the baseline reading (T3), without any deep breathing prior to it. The whole procedure is summarized in Fig. 2.

### EEG and ERP data acquisition

EEG signals were obtained using an NCC Medical 32 Channels Type A Routine EEG System (Model no: Nation 7128W-A32). The electrode cap consists of 32 Ag/AgCl electrodes in accordance with the International 10–20 (site: Fp1, Fp2, AF3, AF4, F7, F3, Fz, F4, F8, FT7, FC3, FC4, FT8, T3, C3, Cz, C4, T4, CP7, CP3, CP4, CP8, P3, Pz, P4, PO3, PO4, T5, O1, Oz, O2, T6). The ground electrode was placed at Fpz while the electrode at Cz serves as the reference. The electrode cap was then connected to Type-A EEG amplifier with a sampling rate of 256 Hz and the signal was transmitted wirelessly and received through a Bluetooth receiver that was connected to a computer. The EEG signals were displayed on the NCC Medical EEG software. The EEG raw data were processed by using FASTER (Nolan, Whelan & Reilly, 2010) which operates as a plug-in in EEGLAB (Lopez-Calderon & Luck, 2014). The high-pass, low-pass, and notch filter frequency were 1 Hz, 30 Hz, and 50 Hz, respectively. The signals were re-referenced to a common average of all the 32 channels and artifactual signals were removed in the channels, epochs, decomposed independent component and single-channel single-epochs. Bad channels were also detected and interpolated. Furthermore, epochs that have an amplitude greater than 75 µV were removed from subsequent analysis as these signals were likely due to movement artifacts. For ERP analysis, only the signals from the midline electrodes (Fz, Cz, and Pz) were analyzed. The EEG raw data were segmented into epochs ranging from 200 ms before the stimulus and 800 ms post-stimulus, with baseline correction from −200 ms to 0 ms (0 ms representing the stimulus onset). Two major components of the ERP were focused on, the N2 (peaks at approximately 100 ms–300 ms after stimulus) and P3 (peaks at approximately 300 ms–600 ms). The time windows for the peak detection were determined from the grand average waveform of the ERP of the Go and NoGo trials. Only correct trials were used for the ERP waveform and the Go and NoGo trials waveforms were obtained separately. An area based measurement for the amplitude was selected to analyze the negative or positive area under the ERP waveform in the two time windows for N2 and P3 as to reduce the effect of latency jittery (Luck, 2014). The peak latency was used to quantify the latency.

### Statistical analysis

The behavioral results from the Go/NoGo task were analyzed using a 4 × 2 repeated ANCOVA with the Group (Con, DB5, DB7, and DB9) as the between-subject factor and the Time (T2 and T3) as the within subject factor. For the ERP result (N2 and P3 amplitudes and latencies), they were analyzed using a 4 × 2  × 3 × 2 repeated ANCOVA with the Group (Con, DB5, DB7, and DB9) as the between-subject factor and Time (T2 and T3), Site (Fz, Cz, and Pz), and Condition (Go and NoGo) as the within subject factors. The respective baseline readings at T1 were used as the covariates for each ANCOVAs. The Greenhouse-Geisser (when ε < 0.75) or Huynh-Feldt (when ε > 0.75) correction was applied when necessary. For any significant finding involving the Group, a planned contrast of comparing the Con to each of the DB groups were done with Bonferroni correction. For the rest of the variables, a pairwise comparisons with Bonferroni correction were used to find the specific changes. A *p* value of <0.05 was considered as statistically significant while a *p* value of <0.10 was reported as a trend. Values are reported in mean and standard deviation in parenthesis.

**Table 2:** The adjusted Go/NoGo overall accuracy, omission error, commission error, Go reaction time, and reaction time variability. The values are expressed in mean and standard deviation in brackets.

	Group	T2	T3
Mean overall accuracy (%)	Con	92.3 (2.8)	92.6 (3.4)
	DB5	93.1 (2.8)	92.3 (3.5)
	DB7	92.4 (2.8)	92.8 (3.4)
	DB9	92.9 (2.8)	92.2 (3.4)
Omission error (%)	Con	1.2 (1.5)	0.2 (0.6)
	DB5	0.5 (1.5)	0.4 (0.6)
	DB7	0.9 (1.5)	0.5 (0.6)
	DB9	0.9 (1.5)	0.4 (0.6)
Comission error (%)	Con	34.3 (10.5)	34.5 (15.0)
	DB5	31.3 (10.7)	35.4 (15.2)
	DB7	35.8 (10.6)	35.2 (15.0)
	DB9	33.3 (10.6)	39.0 (15.1)
Go reaction time (ms)	Con	429.0 (25.1)	427.4 (22.8)
	DB5	430.0 (26.3)	414.8 (23.9)^*^
	DB7	431.6 (25.1)	430.7 (22.8)
	DB9	436.8 (25.3)	412.5 (22.9)^**^
Reaction time variability (ms)	Con	75.5 (14.9)	70.4 (14.5)
	DB5	69.5 (14.9)	67.9 (14.6)
	DB7	70.7 (14.9)	71.0 (14.6)
	DB9	70.4 (14.9)	60.7 (14.6)

**Notes.**

T1 first Go/NoGo task T2 second Go/NoGo task T3 third Go/NoGo task

* ^∗^*p* < 0.05.

** *p* < 0.001 as compared to T2.

**Figure 3:**
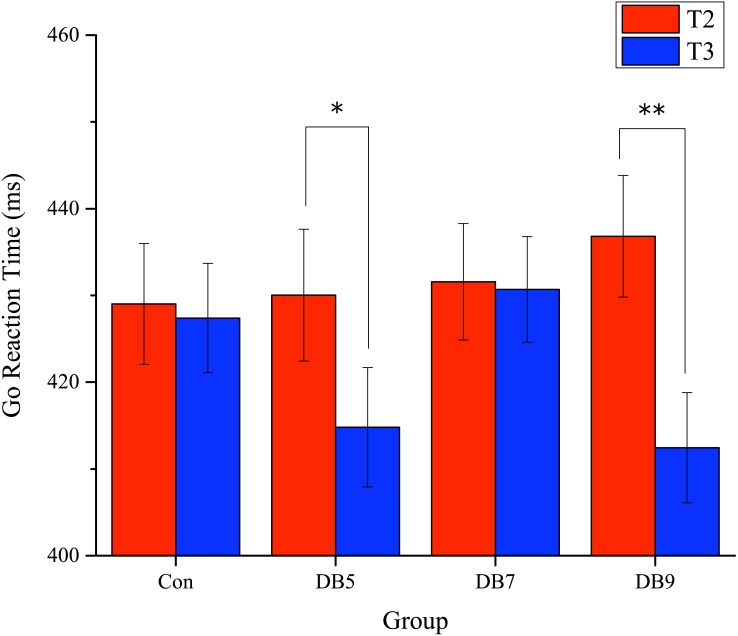
The adjusted mean Go reaction time for T2 and T3. Error bars represent the standard error.^∗^, *p* < 0.05;^∗∗^, *p* < 0.005.

## Results

### Behavioral result

The means and standard deviations of the behavioral data are shown in Table 2. For the Go reaction time (RT), there was a significant Time main effect (*F*(1, 48) = 10.313, *p* = 0.002, ${\eta }_{p}^{2}=0.177$) and a Group × Time interaction (*F*(3, 47) = 3.040, *p* = 0.038, ${\eta }_{p}^{2}=0.162$). Post-hoc analysis showed that the reaction time for T3 was shorter than that of T2 for DB5 and DB9 (DB5: 414.8 (23.9) vs 430.0 (26.3), *p* = 0.039; DB9: 412.5 (22.9) vs 436.8 (25.3), *p* = 0.001), presented in Fig. 3. There was no significance for the Group main effect (*F*(3, 47) = 0.425, *p* = 0.736, ${\eta }_{p}^{2}=0.026$). For OE and RTV, both had a significant Time main effect (*F*(1, 48) = 6.575, *p* = 0.014, ${\eta }_{p}^{2}=0.120$ and *F*(1, 48) = 5.801, *p* = 0.020, ${\eta }_{p}^{2}=0.108$, respectively) with the value at T3 being smaller than that of T2. However, no significance was reached for the Group main effect (OE: *F*(3, 47) = 0.024, *p* = 0.995, ${\eta }_{p}^{2}=0.002$; RTV: (*F*(3, 47) = 0.724, *p* = 0.543, ${\eta }_{p}^{2}=0.044$) and Group × Time interaction (OE: *F*(3, 47) = 0.659, *p* = 0.581, ${\eta }_{p}^{2}=0.040$; RTV: (*F*(3, 47) = 1.668, *p* = 0.187, ${\eta }_{p}^{2}=0.096$). For OA and CE, there were no significant main effects nor interaction.

### ERP data

**Figure 4:**
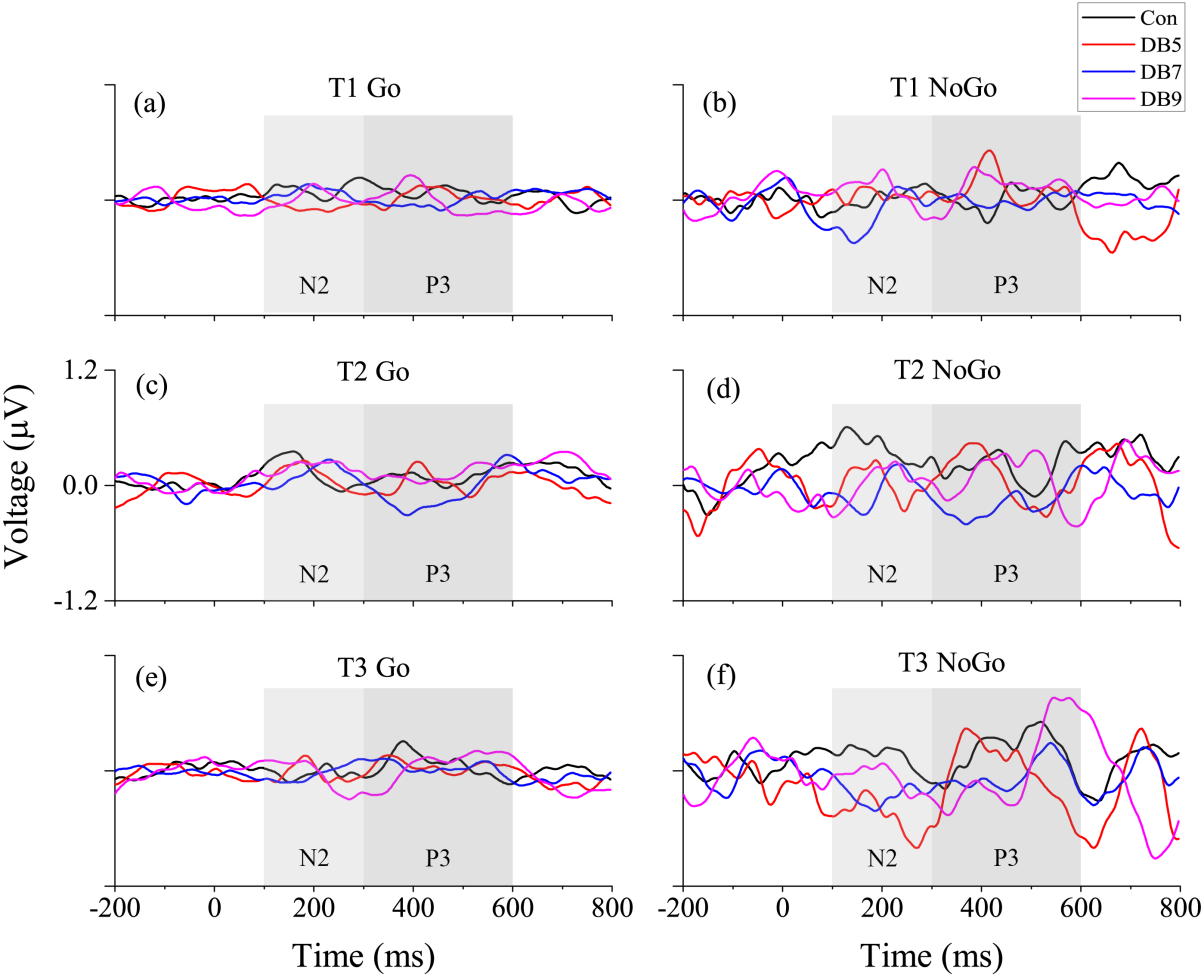
The grand average waveform averaged across the three midline electrodes. (A) T1 Go, (B) T1 NoGo, (C) T2 Go, (D) T2 NoGo, (E) T3 Go, (F) T3 NoGo.

**Figure 5:**
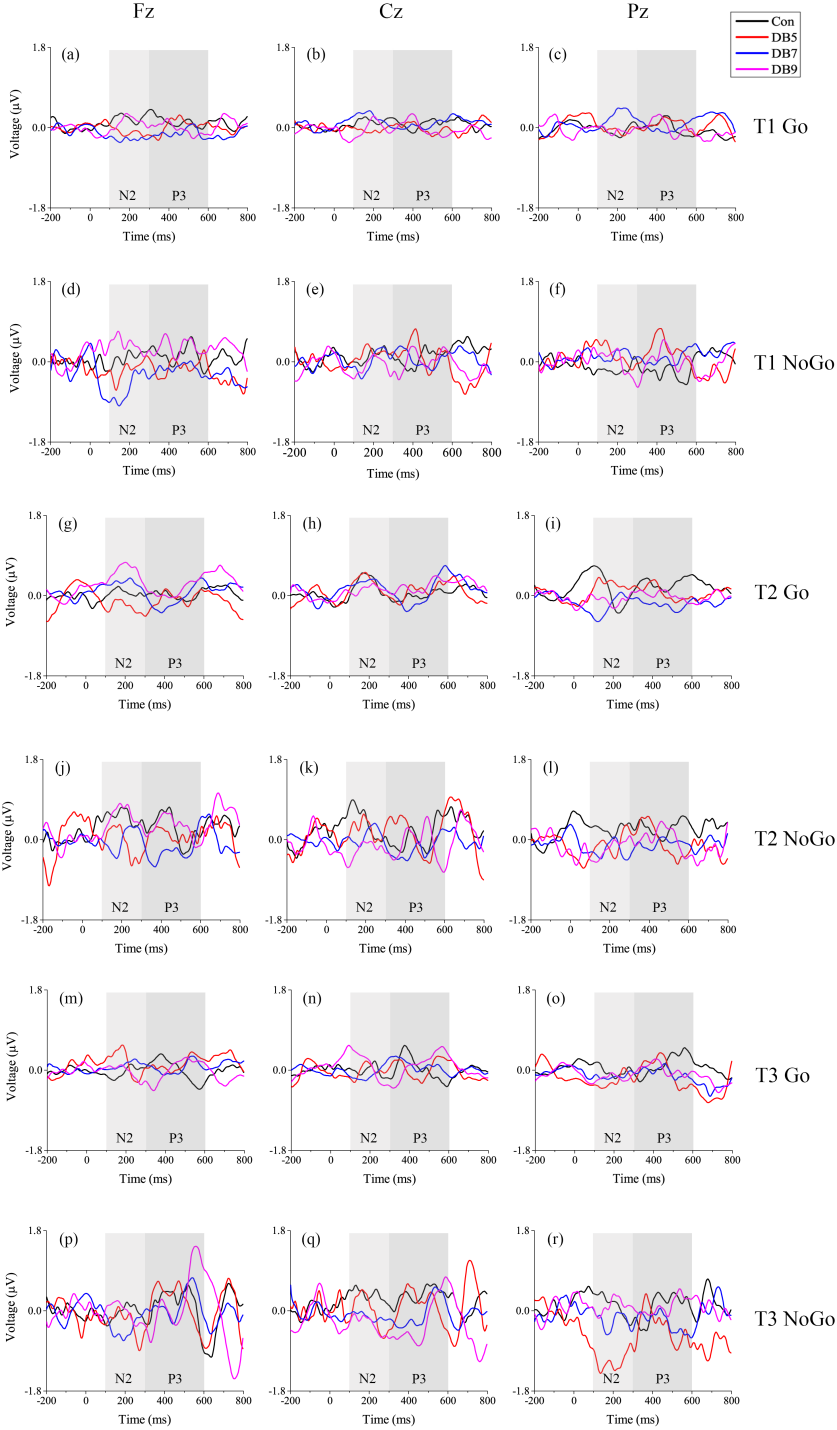
The grand average waveform at each electrode site (Fz, Cz, and Pz). Both Go and NoGo condition ERP waveforms are shown at each Time (T1, T2, and T3) for the four groups.

The ERP data from one of the participants in DB5 was removed from analysis due to too few trials available for averaging (less than one-third of the total available 120 NoGo trials). The number of eligible trials used to obtain the ERP waveform for each group at each time of T1, T2, and T3 is shown in Table 3. The *p* values for each of the main effects and the interactions of the N2 and P3 latency and amplitude are summarized in Table 4. The grand average waveforms for each condition (Go/NoGo) averaged across three midline electrodes (Fz, Cz, and Pz) at T1, T2 and T3 are plotted in Fig. 4 whereas the grand average waveforms for each electrode site individually are plotted in Fig. 5.

**Table 3:** The number of eligible trials used to obtain the ERP waveform for the four groups at T1, T2, and T3. The values are expressed in means and standard deviations in brackets.

Group	Condition	T1	T2	T3
Con	Go	417 (51)	383 (94)	389 (99)
	NoGo	71 (16)	68 (26)	67 (26)
DB5	Go	432 (44)	399 (95)	378 (102)
	NoGo	69 (24)	65 (30)	56 (33)
DB7	Go	405 (86)	421 (36)	410 (66)
	NoGo	73 (19)	74 (19)	71 (23)
DB9	Go	414 (55)	375 (92)	407 (63)
	NoGo	76 (20)	68 (24)	66 (25)

**Notes.**

T1 first Go/NoGo task T2 second Go/NoGo task T3 third Go/NoGo task

**Table 4:** The *p* value for the main effects and interactions in N2 and P3 amplitude and latency. The significant effects/interactions (*p* < 0.05) are bolded while trends (*p* < 0.10) are reported as well.

Effects/interactions	*p* value			
	N2		P3	
	Amplitude	Latency	Amplitude	Latency
T	0.064	ns	ns	ns
S	**0.040**	ns	0.062	ns
C	<**0.001**	ns	<**0.001**	ns
G	ns	0.074	ns	ns
T × S	ns	ns	0.084	ns
T × C	ns	ns	ns	0.051
T × G	ns	ns	ns	**0.045**
S × C	ns	ns	0.059	**0.022**
S × G	ns	ns	ns	ns
C × G	**0.029**	0.090	ns	ns
T × S × C	ns	ns	**0.043**	ns
T × S × G	0.072	ns	ns	ns
T × C × G	**0.040**	0.093	ns	ns
S × C × G	ns	ns	ns	ns
T × S × C × G	ns	ns	ns	ns

**Notes.**

T Time S Site C Condition G Group ns not significant

#### N2

For the N2 amplitude, there was a significant Condition main effect (*F*(1, 46) = 47.423, *p* < 0.001, ${\eta }_{p}^{2}=0.508$), showing the expected larger in the NoGo condition. Besides that, a significant Site main effect (*F*(2, 92) = 3.344, *p* = 0.040, ${\eta }_{p}^{2}=0.068$) was observed but the post-hoc analysis failed to find any significance after Bonferroni correction. Significant Condition × Group interaction (*F*(3, 40) = 3.342, *p* = 0.029, ${\eta }_{p}^{2}=0.200$) and Time × Condition × Group interaction (*F*(3, 40) = 3.048, *p* = 0.040, ${\eta }_{p}^{2}=0.186$) were also evident. Post-hoc analysis showed that the NoGo N2 amplitude was larger at T3 as compared to T2 for the DB5 group (0.166 (0.090) vs 0.096 (0.073), *p* = 0.027) and was larger than the control group at T3 (0.166 (0.090) vs 0.069 (0.087), *p* = 0.040). Several trends were observed as well which included a Time main effect (*F*(1, 46) = 3.596, *p* = 0.064, ${\eta }_{p}^{2}=0.073$) and a Time × Site × Group interaction (*F*(6, 80) = 2.023, *p* = 0.072, ${\eta }_{p}^{2}=0.132$). When inspecting just the main effect of Group, no significance was reached (*F*(3, 40) = 1.236, *p* = 0.309, ${\eta }_{p}^{2}=0.085$). With regards to the latency, there were only three trends involving the Group, namely the Group main effect (*F*(3, 40) = 2.487, *p* = 0.074, ${\eta }_{p}^{2}=0.157$), Condition × Group interaction (*F*(3, 40) = 2.322, *p* = 0.090, ${\eta }_{p}^{2}=0.148$) and Time × Condition × Group interaction (*F*(3, 40) = 2.288, *p* = 0.093, ${\eta }_{p}^{2}=0.146$). The three main effects of Time (*F*(1, 46) = 0.104, *p* = 0.749, ${\eta }_{p}^{2}=0.002$), Site (*F*(2, 92) = 0.614, *p* = 0.543, ${\eta }_{p}^{2}=0.0013$), and Condition (*F*(1, 46) = 0.159, *p* = 0.692, ${\eta }_{p}^{2}=0.003$) were not significant for the N2 latency.

#### P3

For the P3 amplitude, significant Condition main effect (*F*(1, 46) = 41.868, *p* < 0.001, ${\eta }_{p}^{2}=0.476$) and Time × Site × Condition interaction (*F*(2, 92) = 3.261, *p* = 0.043, ${\eta }_{p}^{2}=0.066$) were found, showing that the NoGo P3 having a fronto-central distribution was larger than the Go condition which had a fronto-parietal distribution. Three trends were observed: Site main effect (*F*(2, 92) = 2.873, *p* = 0.062, ${\eta }_{p}^{2}=0.059$), Time ×Site interaction (*F*(2, 92) = 2.546, *p* = 0.084, ${\eta }_{p}^{2}=0.052$) and Site × Condition interaction (*F*(2, 92) = 2.918, *p* = 0.059, ${\eta }_{p}^{2}=0.060$). No main effects of Time (*F*(1, 46) = 0.450, *p* = 0.506, ${\eta }_{p}^{2}=0.010$), Group (*F*(3, 40) = 1.529, *p* = 0.222, ${\eta }_{p}^{2}=0.103$), and interactions involving the Group was observed. As for the latency, there was a significant Time × Group interaction (*F*(3, 40) = 2.930, *p* = 0.045, ${\eta }_{p}^{2}=0.180$) whereby the latency at T3 was shorter than that of T2 for the DB7 group. Besides that, a significant Site × Group interaction (*F*(6, 80) = 3.256, *p* = 0.006, ${\eta }_{p}^{2}=0.196$) was found as well. Post-hoc analysis revealed that at Fz, the latency for the Con group was shorter than DB7 (430.9 (50.5) vs 490.2 (51.2), *p* = 0.018) whereas at Pz, the latency of DB5 was shorter than Con (421.1 (44.4) vs 480.5 (43.1), *p* = 0.006). Lastly, there was also a Time × Condition interaction trend (*F*(1, 46) = 4.001, *p* = 0.051, ${\eta }_{p}^{2}=0.080$). None of the four main effects of Time (*F*(1, 46) = 0.592, *p* = 0.446, ${\eta }_{p}^{2}=0.013$), Site (*F*(2, 92) = 1.024, *p* = 0.363, ${\eta }_{p}^{2}=0.022$), Condition (*F*(1, 46) = 0.105, *p* = 0.747, ${\eta }_{p}^{2}=0.002$), and Group (*F*(3, 40) = 1.034, *p* = 0.388, ${\eta }_{p}^{2}=0.072$) were significant.

## Discussion

This study investigated the effect of different lengths of deep breathing (5 min, 7 min and 9 min) on the behavioral and neurophysiological measurement by using a Go/NoGo paradigm. On the behavioral level in terms of the mean overall accuracy, omission and commission error, Go reaction time, and reaction time variability, there was no difference between groups at T2 or T3. However, for both DB5 and DB9, the Go RT at T3 was shorter than that of their respective T2 value (Table 2). On the neurophysiological measurement of ERP, the NoGo N2 amplitude for the DB5 group was larger than that of Con at T3, indicative of an enhanced conflict monitoring towards the NoGo trials after seven days of practicing deep breathing for 5 min. There was no group effect on the N2 latency as well as for the P3 amplitude. Given that the omission error, reaction time variability, and P3 amplitude were not different between groups after the intervention, it was suggested that deep breathing at six breaths per minute was not able to increase the sustained attention level. However, there was also a Time × Group interaction for the P3 latency (Table 4). At Fz, the latency for Con is shorter than DB7 while the opposite was true at Pz, with the difference now between Con and DB5. Collectively, these results showed that practicing short duration of deep breathing can improve the ability to perceive conflict and possibly increase the cognitive processing speed.

Deep breathing is a common respiration exercise in many mindfulness practices including yoga (Brown & Gerbarg, 2005), tai chi (Shou-Yu & Wen-Ching, 2014), MBSR (Kabat-Zinn, 1982) and many others. This is due to the fact that deep breathing gives a greater sense of body existence, which makes it easy to bring back the attention. Whenever the mind starts to wander, one would need to acknowledge and bring the attention back towards the breathing (Arch & Craske, 2006). In this study, the participants would need to place their attention on the appearing and disappearing petals in the video in order to follow the pre-set breathing frequency of six breaths per minute, along with the instruction of noticing their breath. This very much similar to the instructions given by teachers of mindfulness practices. Following this, literature that reports on mindfulness practices that encompass mindful deep breathing supports the current results of greater conflict monitoring. In a study conducted by Menezes et al. (2013), they evaluated the effects of a six-week-focused meditation training (focusing one’s attention on the breath) on the regulation of emotion and attention on 74 college students and found that there were fewer omission errors in a visual discrimination task for the students in the focused meditation groups compared to the control group. A similar result was shown earlier by Tang et al. (2007) who had investigated a short-term effect of integrative body-mind training (IMBT) on the attention using 40 undergraduate students. IMBT incorporates elements from traditional Chinese medicine practices and contemporary mindfulness practices, in which one of them was the mindful breathing. By the end of the five days of training, the participants have enhanced conflict monitoring as indexed by a better performance in the Attentional Network Task (ANT). Furthermore, studies using ERP to investigate the cognitive process of practicing mindfulness practices have shown a similar increase in the N2 component for people with higher trait mindfulness (Quaglia, Goodman & Brown, 2016), and in adults (Moore et al., 2012) and seniors (Malinowski et al., 2017) as compared to control groups. The discussion above proved that in general, practitioners of mindfulness practices would have a greater conflict monitoring ability, and the current results extended the possibility of achieving the same enhancement simply by performing deep breathing at six breaths per minute every day without any supervision as opposed to the whole set of mindfulness practices.

In addition, studies that investigated the brain activation of mindfulness practices provided further evidence towards the current results. The neural source of the NoGo N2 in a Go/NoGo task was found to be at the anterior cingulate cortex (ACC; Bokura, Yamaguchi & Kobayashi, 2001) and the vast research on the function of the ACC had firmed-up its role in the monitoring of conflict information (De Zubicaray et al., 2000; Braver et al., 2001; Durston et al., 2003). The activation of the ACC will be greater under three categories of situations: response override, selection of equally possible responses and situations involving error (Botvinick, Cohen & Carter, 2004), in which all three involve conflicting information. The practicing of mindfulness practices or meditation is found to have greater activation of the ACC along with several frontal and parietal regions of the brain through several *f*MRI studies (Brefczynski-Lewis et al., 2007; Hölzel et al., 2007; Short et al., 2010; Dickenson et al., 2013). This result implies that mindfulness practitioners are more adept in monitoring and perceiving conflicting information by engaging more attentional resources. This is in line with the state of mindfulness, which entails the need to be aware of one’s thought on the present without any judgment and to bring back one’s mind whenever it is lost. This phenomenon was studied and verified by Hasenkamp et al. (2012) such that the activation of the ACC was the greatest during the awareness-of-mind-wandering process. Relating back to ERP, since the neural source of the N2 component is the ACC, a greater activation of the ACC will inevitably lead to a greater N2 amplitude. Thus, thegreater NoGo N2 amplitude from the current result and hence, greater conflict monitoring may provide evidence that by deep breathing there would be a greater activation of the ACC, just like other mindfulness practice.

Another point that this study addressed was the effects of varying lengths of deep breathing at both the immediate and a follow-up level after practicing for seven days. Current results showed that there was no group difference at the immediate level for both behavioral and neurophysiological results. Even though a relatively small amount of literature has reported the immediate effect following a meditation session, there is evidence showing that the immediate effect in this study would be too small. In a study by Johnson et al. (2013), a single session of mindfulness meditation for 25 min did not lead to greater performance in a battery of cognitive and memory tasks, although it did produce an improvement on the mood states; whereas Polak (2009) had assessed the attentional efficiency of 150 novice meditators assigned to 15 min of mindfulness training, relaxation training or neutral task immediately after the second session. His result revealed that an overall better attention was not achieved by the mindfulness group as compared to the other two groups. Combining the above two studies and the current study, it may seem that a single session of practice is not able to improve the cognitive ability of the participants, even when the practice time is extended up to 25 min. Thus, the maximum deep breathing of 9 min in this study was not able to produce any observable difference between groups. Even though the latter study had evaluated the results at the end of the second session, two days are still considered short compared to longer mindfulness practices that can last up to weeks or even months, and hence, was included as an immediate effect.

**Figure 6:**
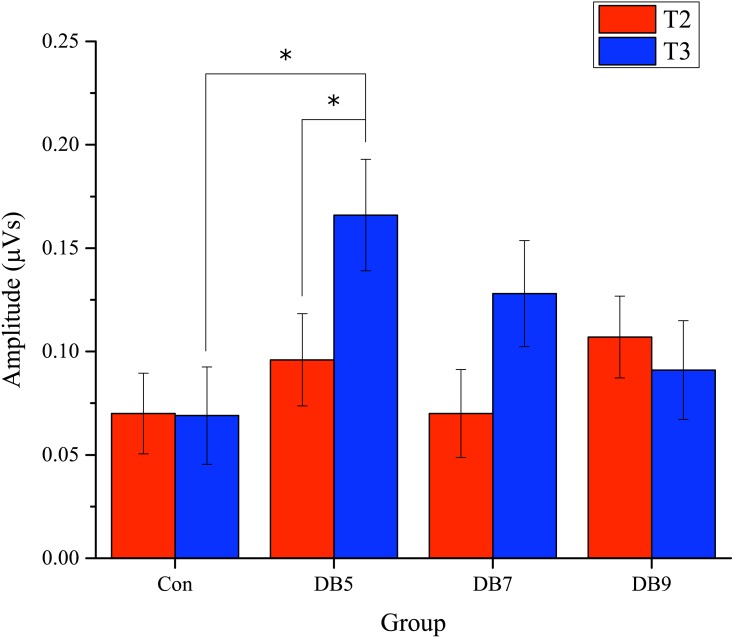
The adjusted amplitude of the NoGo N2 component. The error bars represent the standard error. ^∗^, *p* < 0.05.

As for a follow-up at T3, the result presented here suggests that 5 min of deep breathing was optimum in achieving an enhanced conflict monitoring ability. From Fig. 6, the NoGo N2 amplitude for the DB groups was generally larger than the control group at T3 and the amplitude decreases with increase in the deep breathing duration. This study is one of the few studies that investigated the effect of practice duration. In a preliminary study by Carmody & Baer (2009), they had reviewed the class contact hours of MBSR program and its corresponding effect size on psychological distress. They found that the correlation between the duration and the effect size was not significant and this suggests the fact that a shorter duration of the MBSR program can achieve the same effect. A different study conducted by Prasad et al. (2011) investigating a 5, 15 and 30 min of paced breathing meditation on the stress and quality of life among health care professionals concluded that the duration of practice did not affect the improvement of the stress and quality of life, despite having the 15 min practice generating the greatest improvement. Although the current result is not consistent with the above study, but due to the difference in terms of methodology, study duration and assessment, the effect of the duration of practice on the end results may have been modulated. However, the exact mechanism relating the deep breathing duration to the conflict monitoring and why 5 min is the optimum practice duration still remains unclear. Further imaging studies may provide insight into the mechanism in terms of the change in brain structure and activation achieved by deep breathing.

Lastly, regarding the sustained attention and response inhibition, the current results do not support the hypothesis that deep breathing can increase the sustained attention and response inhibition level. This is evident from the fact that there was no intergroup difference in terms of the behavioral results and the P3 component between the DB groups and the control group at both T2 and T3. The absence of improvements for the behavioral result is in line with previous reports that had investigated mindfulness practices using either a neutral or emotional Go/NoGo task (Hoogland, 2011; Paul et al., 2013; Meland et al., 2015). However, the absence of improvements for the P3 component amplitude (both Go and NoGo condition) is in contrast with previous literature reporting a greater P3 amplitude for practitioners of mindfulness as compared to a control group (Moore et al., 2012; Delgado-Pastor et al., 2013; Schoenberg et al., 2014; Atchley et al., 2016). One possible explanation is that different aspect of the cognitive control enhancements (i.e., conflict monitoring, sustained attention, and respond inhibition) is associated with a particular exercise. From this study, it seems possible that only the conflict monitoring is associated with the deep breathing while the sustained attention and response inhibition are associated with other exercises.

One of the limitations of this study is the small sample size in each group. Further studies should have a larger sample size in order to have a better understanding of deep breathing on the cognitive control. Further, the durations of deep breathing studied here are considered short as compared to the majority of the mindfulness practices. Longer duration of deep breathing comparable to standard mindfulness practices can be investigated; however, one should take caution on the hyperventilation, which was experienced by one of the participants in the DB9 group. In this study, there was no restriction on the method of breathing, but with a suitable breathing method such as the pursed lips method (Prinsloo et al., 2013), the risk of hyperventilation could be reduced. Another possibility is to analyze the data separately according to gender because the underlying brain structure, especially the ACC is different between a male and a female (Yücel et al., 2001).

## Conclusion

In this study involving young adults, an ERP analysis using a video assisted deep breathing was able to enhance the conflict monitoring ability, particularly, during the follow-up session after participants practiced 5 min of video-assisted deep breathing for seven consecutive days. This is clearly shown by the enhanced NoGo N2 amplitude. The observed trend shows that the NoGo N2 amplitude for the deep breathing group during the follow-up session was larger, although only DB5 achieved a statistically significant difference from the control group. As the deep breathing duration increases, the NoGo N2 amplitude reduced. The ANCOVA statistic outcome revealed that there were no effects of deep breathing on the sustained attention and response inhibition at both immediate and follow-up levels of seven days. The results also suggest that 5 min of deep breathing for seven consecutive days seems to be the optimum practice duration as compared to 7 and 9 min of deep breathing.

## Additional Information and Declarations

### Data Availability

The following information was supplied regarding data availability: Cheng, Kok Suen; Chang, Yun Fah; Han, Ray P.S.; Lee, Poh Foong (2017): Raw EEG data for ‘Enhanced conflict monitoring via deep breathing’. figshare.
https://dx.doi.org/10.6084/m9.figshare.5188030.v1.
